# μ-Calpain as a Novel Target for Impairment of Nitric Oxide-Mediated Vascular Relaxation in Diabetes: A Mini Review

**DOI:** 10.4172/1747-0862.1000167

**Published:** 2015-04-04

**Authors:** Raj Kishore, Cynthia Benedict, Zhongjian Cheng

**Affiliations:** Center for Translational Medicine and Department of Pharmacology, Temple University School of Medicine, USA

**Keywords:** μ-Calpain, Endothelial dysfunction, Nitric oxide-mediated vascular relaxation, Diabetes

## Abstract

Diabetes is one of the most prevalent metabolic disorders. In diabetes, incidence of coronary artery diseases and peripheral vascular diseases is increased 2- to 4-fold and 10-fold, respectively, compared to healthy individuals. In spite of extensive studies, the underlying mechanisms of endothelial dysfunction (ED), an early event in the development of vascular diseases, remain incompletely understood in diabetes. This mini-review discusses the role and signaling pathways of calpains - a family of Ca^2+^-sensitive intracellular proteases in nitric oxide (NO)-mediated ED in diabetes. We conclude that activation of calpains, especially μ-calpain, plays an important role in the pathogenesis of NO-mediated ED and inflammatory responses in diabetes which is mainly via endothelial Nitric Oxide Synthase (eNOS) inactivation/degradation in macro- and micro-vasculature. We review existing literature demonstrating that hyperhomocysteinemia, elevated plasma homocysteine level, potentiates hyperglycemia-induced ED via μ-calpain/PKCβ_2_ activation-induced eNOS-pThr497/495 and eNOS inactivation. μ-calpain may be a critical therapeutic target for NO-mediated ED in diabetes.

## Introduction

Diabetes, one of the most prevalent metabolic disorders, is estimated to affect 400 million or 4.4% of population worldwide in the next 20 years [1,2]. Vascular abnormalities are the major contributor to the progression of diabetes which is associated with complications often linked to the increased morbidity and mortality. In diabetic patients, coronary artery disease and peripheral vascular diseases are increased to 2- to 4-fold and 10-fold, respectively, compared to non-diabetic individuals.

The endothelium is a monolayer of endothelial cells (ECs) lining the lumen of all blood vessels and functions as a protective biocompatible barrier between tissues and circulating blood. In human, there is a range of 1–6×10^13^ ECs covering the surface area of more than 1,000 square meters of endothelium [3,4]. The endothelium plays a key role in the control of vasomotor tone and organ perfusion, and contributes to regulation of arterial blood pressure, by releasing vasodilator substances and vasoconstrictor substances [5,6]. Endothelial dysfunction (ED) is a systemic pathological condition, which can be characterized by an impairment of endothelium-dependent vasodilatation. ED is an early event in the development of cardiovascular disease prior to any visible morphological changes in endothelium. Recently, ED has been linked to endothelial activation [7] including increased interaction between inflammatory cells/factors (leukocytes, intercellular adhesion molecule, vascular cell adhesion molecule 1 and selectins) and endothelial cells, and albumin leakage. Numerous studies have showed that the endothelial function is impaired in both diabetic patients and animals. Therefore, understanding the underlying mechanisms of ED may provide novel promising therapeutic strategies for the treatment of cardiovascular diseases in diabetes.

Calpains are a family of cytosolic calcium-dependent cysteine-proteases which tightly regulate their respective substrates through limited proteolytic cleavage. Elevated calpains expression or activity has been found in patients [8] and experimental animals [9,10] with diabetes. Inhibition of calpains activity rescues hyperglycemia-mediated vascular injury, inflammation and ED [9,10].

Endothelial nitric oxide (NO) was first recognized as a major vasodilator involved in control of vasomotor function and local blood flow. Endothelial NO is mainly generated by constitutively active endothelial NO synthase (eNOS), an essential enzyme responsible for vascular homeostasis. Loss of NO bioavailability as a result of decrease of eNOS activity has been speculated to play an essential role in the pathogenesis of ED. In this review, we summarize the current understanding of the role of calpains activation and signaling pathways in NO-mediated ED in diabetes.

## Calpain Family

There are currently 15 known human calpain isoform genes. Members of the calpain family are believed to function in various biological processes, including integrin-mediated cell migration, cytoskeletal remodeling, cell differentiation and apoptosis [11]. Activation of calpain has been implicated in the acute and chronic hyperglycemia (HG)-induced ED [9,10], platelet hyperaggregability [12], neurovascular dysfunction [13] and cardiomyocyte apoptosis [12] in diabetes. Within the calpain family, micro (μ)- and milli (m)-calpain are the two most well characterized isoforms. μ- and m-calpain are ubiquitously expressed in mammals, and are activated by micro-and millimolar calcium concentrations in vitro, respectively [11]. Both proteins are heterodimers composed of a large 78–80 kDa catalytic subunit and a common small 29 kDa regulatory subunit [14]. The large subunit comprises four domains (dI-dIV), whereas the small subunit has two domains (dV-dVI). When calpains are activated, they undergo autoproteolysis which removes N-terminal in dI (NT, 27 and 19 amino acids from large subunit (catalytic subunit) of μ- and m-calpain, respectively) [14]. Thus lowers levels of calpain large subunit NT indicates higher degrees of proteolytic activity of calpain [1,15]. Total μ- and m-calpain content was quantified using a primary antibody against the stable domain IV of the large subunit, which recognizes both unautolyzed and autolyzed μ- and m-calpain [1,15]. Previously, it was considered that the calcium influxes are mainly responsible for the activation of calpains. However, the calcium concentrations required for its activation in vitro are much higher compare to that in the physiological condition (100 nM to 10 μM) and not consistent with cell survival. Several mechanisms have been suggested for calpains activation in the presence of lower calcium concentration, such as mitochondrial translocation of calpains via increasing mitochondrial oxidative stress [16] and posttranslational modifications of calpains by kinases thus indirectly activate calpains by increasing its sensitivity to calcium [17]. Activation of μ-calpain is also regulated by its membrane localization and by its binding to phosphatidylinositol [18]. Moreover, Na^+^/H^+^ exchanger is required in hyperglycemia-induced calpains activation [19].

Both μ- and m-calpain are specifically countered by the endogenous calpain inhibitor, calpastatin [11]. Several substrates for calpains have been identified, including cytoskeletal proteins, membrane receptors, protein kinases, and transcription factors [11].

## Calpains in Diabetes

Calpain-10 is the first calpain gene identified in diabetes. The polymorphism of calpain-10 has been linked and associated with diabetes susceptibility, glucose homeostasis, insulin secretion and insulin activation [20,21]. Decreased calpain-3 expression in skeletal muscle is associated with obesity and insulin resistance and linked to diabetes mellitus [22].

The μ-calpain (calpain-1) is activated in diabetes. Activation of μ-calpain has been suggested to be linked to impairment of glucose transporter turnover [23] and ED [9,10,1,24]. Acute exposure of mouse pancreatic islets to μ-calpain decreased insulin-stimulated glucose uptake into adipocytes, skeletal muscle, and glycogen synthesis in muscle. Inhibition of μ-calpain by μ-calpain antisense oligonucleotides or siRNA rescued high glucose-induced ED and vascular inflammation in the micro- and macro-vasculature [9,10,1,24].

Taken together, calpains, especially calpain-10 and −3, and μ-calpain play an important role in the pathogenesis of diabetes, such as insulin resistance, insulin secretion, glycogen synthesis, glucose transporter turnover and ED. Different calpain seem to serve unique roles in the pathogenesis of diabetes, thus discovery of selective calpain inhibitors are critically for the treatment of cardiovascular complications in diabetes.

## Calpain Activation in ECs

**Figure 1:**
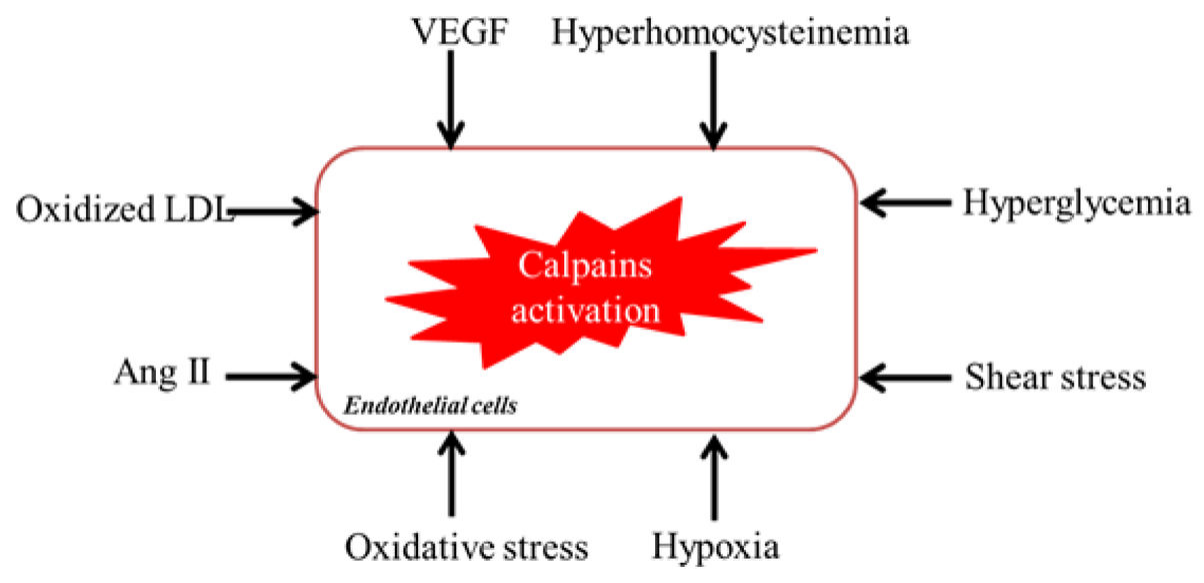
Schematic representation of the risk factors for calpains activation in the ECs. Ang II, angiotensin II; LDL, low density lipoprotein; VEGF, vascular endothelial growth factor.

Both μ- and m-calpain are expressed in vascular cells, including ECs and vascular smooth muscle cells (VSMCs). The role of calpains in regulation of EC functional properties has been extensively examined. Calpains act on ECs, thus maintaining vascular physiological integrity [25–27]. Whereas, over activation of calpains appears to play an important role in pathogenesis of angiogenesis [11,28], ED [9,10,24,29] and wound healing [30]. Many factors have been suggested to induce calpains activation in ECs (Figure 1). Vascular endothelial growth factor increased μ-calpain activity in both human microvascular and bovine aortic endothelial cells [11,31]. Shear stress increased μ-calpain activity in mouse aortic and human umbilical vein endothelial cells (HUVECs) [11,26,32]. Angiotensin II induces μ-calpain activation in the endothelia cells of mouse postcapillary venules [29]. Hypoxia induces calpains activation in porcine pulmonary artery ECs [33]. Moreover, oxidized LDL enhanced μ-calpain activity in HUVECs [34]. Antioxidants reduced μ-calpain but not m-calpain activity in mouse pulmonary microvascular endothelial cells. High glucose increases μ-calpain activity in HVECs [35] and mouse micro- and macro-vascular ECs [9,10,15,24].

Hyperhomocysteinemia (HHcy) - elevated plasma homocysteine (Hcy) concentration has been considered as an independent risk factor for the development of ED [6,15,36]. Recent studies shown a high prevalence of HHcy in patients with diabetes and that plasma concentration of Hcy is positively correlated with cardiovascular mortality and morbidity in diabetes [37]. However the interaction between HHcy and diabetes and the role of HHcy/diabetes on the pathogenesis of cardiovascular diseases remains incompletely understood. We and other groups have shown that HHcy increased μ-calpain activity in micro- and macro-vascular ECs [15,16]. Calpain activity is increased in mice with HHcy and cultured rat heart microvascular ECs [16,38]. Moreover, HHcy induces the translocation of active μ-calpain from cytosol to mitochondria, leading to increased intramitochondrial oxidative stress in cultured rat heart microvascular endothelial cells [16]. We recently studied the effect of HHcy on hyperglycemia-induced calpains activation [15]. We found that D-Hcy (500 μM) for 48 hours potentiated D-glucose (25 mM)-induced calpains activation in both human and mouse aortic endothelial cells [15]. Moreover, μ-calpain siRNA significantly inhibited HHcy/hyperglycemia-induced calpains activity. By our knowledge, we are the first to report that HHcy potentiates hyperglycemia-induced μ-calpain activation in macro-vascular ECs [15]. We believe that our findings will provide fundamental insights for the prevention and treatment of cardiovascular diseases in patients with diabetes and HHcy.

## Calpains-induced ED in Diabetes

In response to pathophysiological stimulation, the endothelium maintains vascular hemostasis by releasing vasodilator substances, such as nitric oxide (NO), prostacyclin (PGI_2_) and endothelium-derived hyperpolarizing factor (EDHF), and vasoconstrictor substances, such as angiotension II, endothelin-1, thromboxane A_2_, and prostaglandin H_2_ [5,6]. ED is characterized by and often defined as a reduced response to an endothelium-dependent vasodilator substances, such as acetylcholine or bradykinin, or to flow-mediated vasodilatation [39].

Therefore, understanding the effect of calpains on the vasodilator substances and underlying mechanisms could provide therapeutic insight in the prevention and treatment of ED in diabetes. In fact, accumulative evidence showed that inhibition of calpains rescued ED and prevented interaction between inflammatory cells (leukocytes)/factors (intercellular adhesion molecule-1 and vascular cell adhesion molecule-1) with ECs in diabetes (Table 1) [9,10,13,15,24,40,41]. However the underlying mechanisms remain unclear.

Effects of calpains activation on eNOS expression/activity and eNOS signaling pathways have been extensively studied. NO production in ECs is modulated through calpains-induced proteolysis of eNOS-associated proteins, such as heat shock proteins 90 (hsp90) [42], caveolin [42], eNOS itself [31,34], Akt [43] or interruption of Akt, hsp90 and eNOS binding [9,10,43] or regulation of PI_3_K/AMPK signaling [31]. Activation of calpains also leads to disruption of eNOS localization by caveolin-3 breakdown in caveolae structures thus induces aberrant eNOS uncoupling [44].

Protein kinase C (PKC) is an important signaling molecule associated with ED in diabetes. Activation of endothelial PKC induces ED in diabetes via regulation of vasodilators and vasoconstrictors [45,46]. Within the PKC family, PKCβ_2_ has received much attention since it was first shown to be preferentially in diabetic vascular tissue [47]. PKCβ_2_ activation mediates HG-induced microvascular inflammation [48] and cardiomyocyte apoptosis [12]. Originally, PKC was found as a substrate of the calpains family which can be cleaved and activated by calpains activation [49,50].

Recent studies suggested that calpains can also serve as a downstream target of PKC signaling [51]. PKC inhibitor BIM-1 decreases calpains activity in mouse microvascular endothelial cells under hyperglycemia condition [48]. Nevertheless, we recently showed that both μ-calpain and PKCβ_2_ inhibition by pharmacological inhibitors or gene silencing approaches rescued HHcy/hyperglycemia-induced eNOS-pThr495 [15]. We demonstrated that HHcy potentiated hyperglycemia-induced ED via μ-calpain/PKCβ_2_ activation-mediated eNOS-pThr49, eNOS inactivation and NO reduction [15].

PGI_2_ is generated from arachidonic acid by cyclooxygenase (COX) including COX-1 and COX-2. PGI_2_ mediates endothelium-dependent vascular relaxation via PGI_2_ receptors (IP) and protects vessels from the development of diseases [52]. Decreased production of PGI_2_ has been suggested to cause an increased incidence of cardiovascular events [53].

In diabetes, PGI_2_ may also act on thromboxane prostanoid receptor (TP) on smooth muscle to mediate vasoconstriction and it functions as an endothelium-dependent contracting factor [54]. COX-2 has been commonly considered a major source of endothelial PGI_2_ synthesis [53]. The role of calpain on the regulation of COX-2 in diabetes remains unclear. Calpain/cathepsin protease inhibitors suppressed cleavage of COX-2 in human synovial fibroblasts [55]. Calpain inhibitor PD150606 dose-dependently increased lipopolysaccharide-induced COX-2 in murine aortic endothelial cells (MAEC), which was dose-dependently degraded by porcine μ-calpain [56].

Moreover, titanium particles stimulate COX-2 expression in fibroblasts via calpain-induced degradation of IκB and activation of NF-κB [57]. Thus, studies on the role of calpain activation on the impairment of PGI_2_-mediated endothelium-dependent vascular relaxation in diabetes are needed.

Cumulative evidence is mounting that EDHF is a major determinant of vascular tone in small resistance vessels. In spite of numerous studies, the nature of EDHF is still not entirely elucidated. The endothelium-mediated relaxation, which is resistant to eNOS and COX inhibition, is thought to be mediated by EDHF. Current evidence suggests that EDHF-mediated responses are initiated by activation of endothelial K^+^ channels (Kca), thus posttranscriptional modification of KCa is suggested to be involved in EDHF-mediated ED under certain disease conditions [6,36,58]. We have found that EDHF-mediated endothelium-dependent vascular relaxation was impaired in the mesenteric artery of diabetic Goto-Kakizaki rats and this relaxation was aggravated by high-salt diet [59]. Moreover, we reported that HHcy impaired EDHF-mediated ED in mouse small mesenteric artery by oxidation and tyrosine-nitration of small and intermediate conductance Kca [36]. Role of calpain on the regulation of Kca in ECs has not been studied under diabetic condition yet. A recent study showed that over expression of endogenous calpain inhibitor calpastatin improved EDHF-mediated ED in the aorta of streptozotocin (STZ)-treated mice, suggesting that activation of calpains may also trigger ED in diabetes via regulation of EDHF. Further studies on the effects of calpain activation on the regulation of EDHF-mediated endothelial dysfunction in diabetes are warranted.

## Conclusions

**Figure 2:**
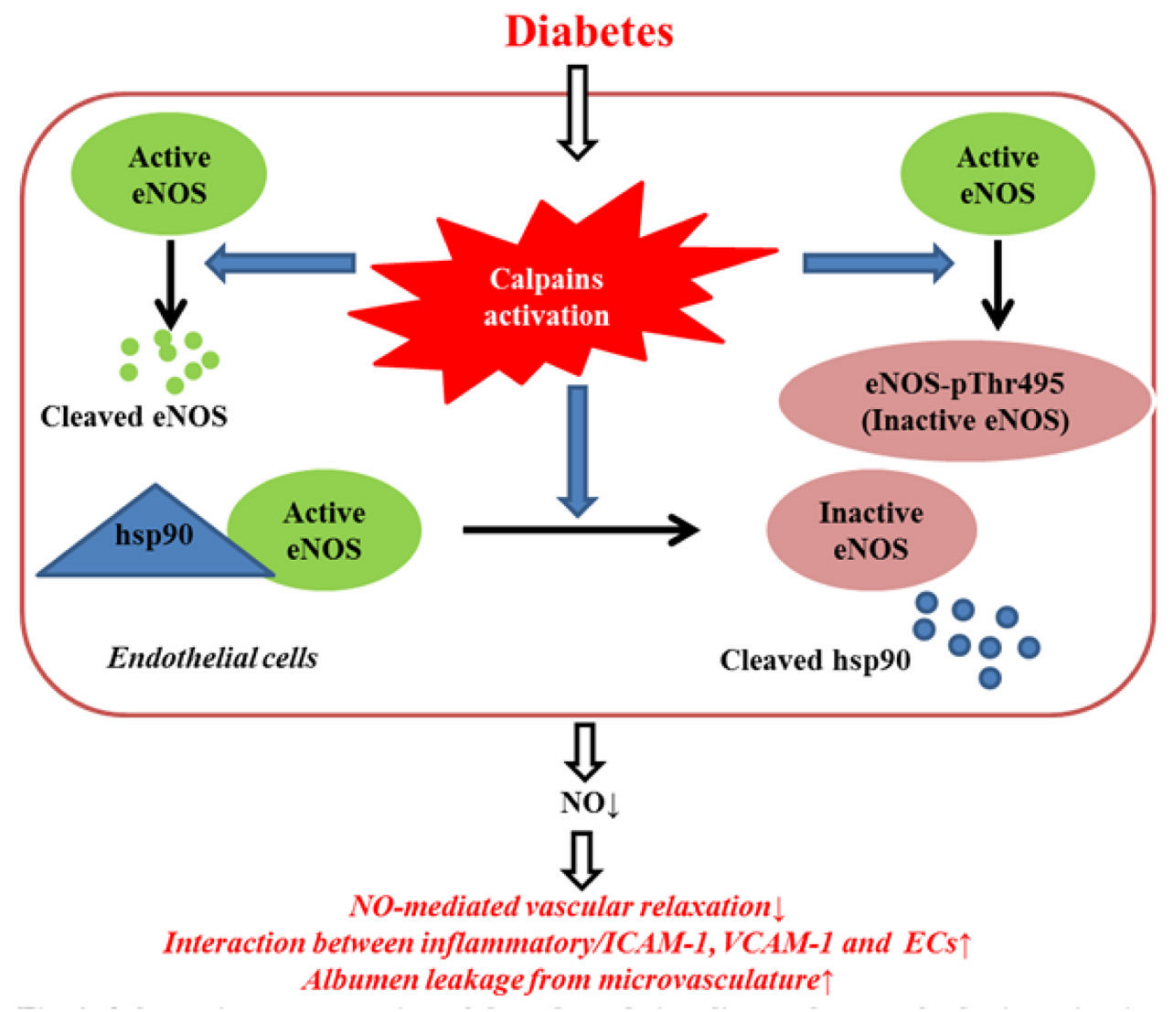
Schematic representation of the role and signaling pathways of calpain activation in NO-mediated ED in diabetes. eNOS, endothelial nitric oxide synthase; ECs, endothelial cells; eNOS-pThr495/497, phosphorylation of eNOS at threonine 495/497; hsp90, ICAM-1, intercellular adhesion molecule 1; hsp90, heat shock proteins 90; NO, nitric oxide; VCAM-1, vascular cell adhesion molecule 1.

Our study and data from other laboratories support the concept that activation of calpains, especially activation of μ-calpain, is causally linked to NO-mediated ED in diabetes. Activation of μ-calpain decreases NO bioavailability by decreasing the interaction between hsp90 and eNOS, increasing eNOS degradation and eNOS-pThr495 (Figure 2). Inhibition of calpains rescued hyperglycemia-induced NO-mediated ED and inflammatory responses in micro- and macro-vasculature. We demonstrate that HHcy potentiates μ-calpain activation thus aggravating ED in diabetes. Given the very recent study reporting that overexpression of calpastatin improved EDHF-mediated ED in the aorta of mice with hyperglycemia [41]. Studies are warranted elucidating this therapeutically useful pathway because the role of activation of calpains on PGI2 and EDHF in diabetes remains unclear.

## Figures and Tables

**Table 1:** Effects of calpains inhibition on ED in diabetic animal models.

Procedure for calpains inhibition	Animal	Vessels	Effective dose	Outcomes	Ref.
MDL-28170	STZ-treated mouse	Thoracic aorta	2 mg/kg/day, i.p. 2 weeks	NO-mediated vascular relaxation to ACh↑	[15]
MDL-28170, ALLM	STZ-treated mouse	Thoracic aorta	Aorta were preincubated with 20 μM of MDL-28170, ALLM or calpeptin for 1h, *ex vivo*	NO-mediated vascular relaxation to ACh↑	[15]
siRNA against μ-calpain	C57BL/6J mouse	Thoracic aorta	Vessels treated with D-glucose and μ-calpain siRNA for 72h, *in vitro*	NO-mediated vascular relaxation to ACh↑	[15]
ZLLal	C57BL6J mouse injected with D-glucose	Mesenteric artery	18 μg/kg/day, i.p., one time	Leukocyte-endothelium interactions↓, NO production↑	[10]
ZLLal	Zucker diabetic fatty rat	Mesenteric artery	27 μg/kg/day, i.p., 5 days	Leukocyte-endothelium interactions↓, NO production↓	[9]
ZLLal and μ-calpain antisense oligonucleotides	Zucker diabetic fatty rat	Mesenteric artery	ZLLal: 27μg/kg/day, i.p., 4 days μ-calpain antisense oligonucleotides: 1mg/kg/day, i.p., 4 days	Microvascular albumin leakage↓	[24]
Over-expression of calpastatin	Calpastatin transgenic/STZ-treated, calpastatin transgenic/db/db, and calpastatin transgenic/OVE26 mouse	Aorta		NO- and EDHF-mediated vascular relaxation to ACh↑	[41]
ZLLal PD150606	STZ-treated rats	Mesenteric post - capillary venules	ZLLal: 27 μg/kg/day, i.p., 4 days PD150606: 1mg/kg/day, i.p., 4 days	Leukocyte-endothelium interaction↓	[29]
MDL-28170	C57BL6J mouse	Aorta	Aorta were treated with heavily oxidized’ glycated LDL and MDL-28170 (20 μM) for 6h, *in vitro*	Vascular relaxation to ACh↑	[40]
A-705253	STZ-treated mouse	Cavernosa	30 mg/kg/day, 2 weeks	Cavernosa relaxation to ACh↑	[13]

## References

[1] Gregg EW, Li Y, Wang J, Burrows NR, Ali MK (2014). Changes in diabetes-related complications in the United States, 1990–2010. N Engl J Med.

[2] Unwin N, Gan D, Whiting D (2010). The IDF Diabetes Atlas: providing evidence, raising awareness and promoting action. Diabetes Res Clin Pract.

[3] Jaffe EA (1987). Cell biology of endothelial cells. Hum Pathol.

[4] Mai J, Virtue A, Shen J, Wang H, Yang XF (2013). An evolving new paradigm: endothelial cells--conditional innate immune cells. J Hematol Oncol.

[5] Furchgott RF, Zawadzki JV (1980). The obligatory role of endothelial cells in the relaxation of arterial smooth muscle by acetylcholine. Nature.

[6] Cheng Z, Yang X, Wang H (2009). Hyperhomocysteinemia and Endothelial Dysfunction. Curr Hypertens Rev.

[7] Shao Y, Cheng Z, Li X, Chernaya V, Wang H (2014). Immunosuppressive/anti-inflammatory cytokines directly and indirectly inhibit endothelial dysfunction- a novel mechanism for maintaining vascular function. J Hematol Oncol.

[8] Ling C, Groop L, Guerra SD, Lupi R (2009). Calpain-10 expression is elevated in pancreatic islets from patients with type 2 diabetes. PLoS One.

[9] Stalker TJ, Gong Y, Scalia R (2005). The calcium-dependent protease calpain causes endothelial dysfunction in type 2 diabetes. Diabetes.

[10] Stalker TJ, Skvarka CB, Scalia R (2003). A novel role for calpains in the endothelial dysfunction of hyperglycemia. FASEB J.

[11] Goll DE, Thompson VF, Li H, Wei W, Cong J (2003). The calpain system. Physiol Rev.

[12] Randriamboavonjy V, Pistrosch F, Bolck B, Schwinger RH, Dixit M (2008). Platelet sarcoplasmic endoplasmic reticulum Ca2+-ATPase and mu-calpain activity are altered in type 2 diabetes mellitus and restored by rosiglitazone. Circulation.

[13] Nangle MR, Cotter MA, Cameron NE (2006). The calpain inhibitor, A-7052, corrects penile nitrergic nerve dysfunction in diabetic mice. Eur J Pharmacol.

[14] Li H, Thompson VF, Goll DE (2004). Effects of autolysis on properties of mu- and m-calpain. Biochim Biophys Acta.

[15] Cheng Z, Jiang X, Pansuria M, Fang P, Mai J (2015). Hyperhomocysteinemia and Hyperglycemia Induce and Potentiate Endothelial Dysfunction via Î¼-Calpain Activation. Diabetes.

[16] Moshal KS, Singh M, Sen U, Rosenberger DS, Henderson B (2006). Homocysteine-mediated activation and mitochondrial translocation of calpain regulates MMP-9 in MVEC. American journal of physiology Heart and circulatory physiology.

[17] Glading A, Bodnar RJ, Reynolds IJ, Shiraha H, Satish L (2004). Epidermal growth factor activates m-calpain (calpain II), at least in part, by extracellular signal-regulated kinase-mediated phosphorylation. Mol Cell Biol.

[18] Leloup L, Shao H, Bae YH, Deasy B, Stolz D (2010). m-Calpain activation is regulated by its membrane localization and by its binding to phosphatidylinositol, 5-bisphosphate. J Biol Chem.

[19] Wang S, Peng Q, Zhang J, Liu L (2008). Na+/H+ exchanger is required for hyperglycaemia-induced endothelial dysfunction via calcium-dependent calpain. Cardiovascular research.

[20] Sreenan SK, Zhou YP, Otani K, Hansen PA, Currie KP (2001). Calpains play a role in insulin secretion and action. Diabetes.

[21] Logie LJ, Brown AE, Yeaman SJ, Walker M (2005). Calpain inhibition and insulin action in cultured human muscle cells. Mol Genet Metab.

[22] Walder K, McMillan J, Lapsys N, Kriketos A, Trevaskis J (2002). Calpain 3 gene expression in skeletal muscle is associated with body fat content and measures of insulin resistance. Int J Obes Relat Metab Disord.

[23] Otani K, Han DH, Ford EL, Garcia-Roves PM, Ye H (2004). Calpain system regulates muscle mass and glucose transporter GLUT4 turnover. J Biol Chem.

[24] Scalia R, Gong Y, Berzins B, Zhao LJ, Sharma K (2007). Hyperglycemia is a major determinant of albumin permeability in diabetic microcirculation: the role of mu-calpain. Diabetes.

[25] Miyazaki T, Koya T, Kigawa Y, Oguchi T, Lei XF (2013). Calpain and atherosclerosis. J Atheroscler Thromb.

[26] Miyazaki T, Honda K, Ohata H (2010). m-Calpain antagonizes RhoA overactivation and endothelial barrier dysfunction under disturbed shear conditions. Cardiovasc Res.

[27] Gonscherowski V, Becker BF, Moroder L, Motrescu E, Gil-Parrado S (2006). Calpains: a physiological regulator of the endothelial barrier?. Am J Physiol Heart Circ Physiol.

[28] Kang H, Kwak HI, Kaunas R, Bayless KJ (2011). Fluid shear stress and sphingosine 1-phosphate activate calpain to promote membrane type 1 matrix metalloproteinase (MT1-MMP) membrane translocation and endothelial invasion into three-dimensional collagen matrices. J Biol Chem.

[29] Scalia R, Gong Y, Berzins B, Freund B, Feather D (2011). A novel role for calpain in the endothelial dysfunction induced by activation of angiotensin II type 1 receptor signaling. Circ Res.

[30] Qiu K, Su Y, Block ER (2006). Use of recombinant calpain-2 siRNA adenovirus to assess calpain-2 modulation of lung endothelial cell migration and proliferation. Mol Cell Biochem.

[31] Youn JY, Wang T, Cai H (2009). An ezrin/calpain/PI3K/AMPK/eNOSs1179 signaling cascade mediating VEGF-dependent endothelial nitric oxide production. Circ Res.

[32] Miyazaki T, Honda K, Ohata H (2007). Requirement of Ca2+ influx- and phosphatidylinositol 3-kinase-mediated m-calpain activity for shear stress-induced endothelial cell polarity. Am J Physiol Cell Physiol.

[33] Zhang J, Patel JM, Block ER (1998). Hypoxia-specific upregulation of calpain activity and gene expression in pulmonary artery endothelial cells. Am J Physiol.

[34] Dong Y, Wu Y, Wu M, Wang S, Zhang J (2009). Activation of protease calpain by oxidized and glycated LDL increases the degradation of endothelial nitric oxide synthase. J Cell Mol Med.

[35] Chen YY, Chen J, Zhou XM, Meng XH, Jiang JP (2012). Puerarin protects human umbilical vein endothelial cells against high glucose-induced apoptosis by upregulating heme oxygenase-1 and inhibiting calpain activation. Fundamental & clinical pharmacology.

[36] Cheng Z, Jiang X, Kruger WD, Pratico D, Gupta S (2011). Hyperhomocysteinemia impairs endothelium-derived hyperpolarizing factor-mediated vasorelaxation in transgenic cystathionine beta synthase-deficient mice. Blood.

[37] Hofmann MA, Kohl B, Zumbach MS, Borcea V, Bierhaus A (1998). Hyperhomocyst(e)inemia and endothelial dysfunction in IDDM. Diabetes Care.

[38] Hamelet J, Couty JP, Crain AM, Noll C, Postic C (2009). Calpain activation is required for homocysteine-mediated hepatic degradation of inhibitor I kappa B alpha. Mol Genet Metab.

[39] Ding H, Triggle CR (2010). Endothelial dysfunction in diabetes: multiple targets for treatment. Pflugers Arch.

[40] Dong Y, Wu Y, Wu M, Wang S, Zhang J (2009). Activation of protease calpain by oxidized and glycated LDL increases the degradation of endothelial nitric oxide synthase. J Cell Mol Med.

[41] Chen B, Zhao Q, Ni R, Tang F, Shan L (2014). Inhibition of calpain reduces oxidative stress and attenuates endothelial dysfunction in diabetes. Cardiovasc Diabetol.

[42] Bhuiyan MS, Shioda N, Fukunaga K (2009). Chronic beta-AR activation-induced calpain activation and impaired eNOS-Akt signaling mediates cardiac injury in ovariectomized female rats. Expert Opin Ther Targets.

[43] Smith IJ, Dodd SL (2007). Calpain activation causes a proteasome-dependent increase in protein degradation and inhibits the Akt signalling pathway in rat diaphragm muscle. Exp Physiol.

[44] Han F, Lu YM, Hasegawa H, Kanai H, Hachimura E Inhibition of dystrophin breakdown and endothelial nitric-oxide synthase uncoupling accounts for cytoprotection by 3-[2-[4-(3-chloro-2-methylphenyl)-1-piperazinyl]ethyl]-,6-dimethoxy-1-(4-imidazo lylmethyl)-1H-indazole dihydrochloride 3.5 hydrate (DY-9760e) in left ventricular hypertrophied Mice. J Pharmacol Exp Ther.

[45] Rask-Madsen C, King GL (2013). Vascular complications of diabetes: mechanisms of injury and protective factors. Cell Metab.

[46] Brustolin S, Giugliani R, Felix TM (2010). Genetics of homocysteine metabolism and associated disorders. Brazilian journal of medical and biological research = Revista brasileira de pesquisas medicas e biologicas/Sociedade Brasileira de Biofisica.

[47] Inoguchi T, Battan R, Handler E, Sportsman JR, Heath W (1992). Preferential elevation of protein kinase C isoform beta II and diacylglycerol levels in the aorta and heart of diabetic rats: differential reversibility to glycemic control by islet cell transplantation. Proceedings of the National Academy of Sciences of the United States of America.

[48] Smolock AR, Mishra G, Eguchi K, Eguchi S, Scalia R (2011). Protein kinase C upregulates intercellular adhesion molecule-1 and leukocyte-endothelium interactions in hyperglycemia via activation of endothelial expressed calpain. Arteriosclerosis, thrombosis, and vascular biology.

[49] Inoue M, Kishimoto A, Takai Y, Nishizuka Y (1977). Studies on a cyclic nucleotide-independent protein kinase and its proenzyme in mammalian tissues. II. Proenzyme and its activation by calcium-dependent protease from rat brain. The Journal of biological chemistry.

[50] Takai Y, Yamamoto M, Inoue M, Kishimoto A, Nishizuka Y (1977). A proenzyme of cyclic nucleotide-independent protein kinase and its activation by calcium-dependent neutral protease from rat liver. Biochemical and biophysical research communications.

[51] Xu L, Deng X (2006). Protein kinase Ciota promotes nicotine-induced migration and invasion of cancer cells via phosphorylation of micro- and m-calpains. J Biol Chem.

[52] Bunting S, Gryglewski R, Moncada S, Vane JR (1976). Arterial walls generate from prostaglandin endoperoxides a substance (prostaglandin X) which relaxes strips of mesenteric and coeliac ateries and inhibits platelet aggregation. Prostaglandins.

[53] Grosser T, Yu Y, Fitzgerald GA (2010). Emotion recollected in tranquility: lessons learned from the COX-2 saga. Annu Rev Med.

[54] Zhu N, Liu B, Luo W, Zhang Y, Li H (2014). Vasoconstrictor role of cyclooxygenase-1-mediated prostacyclin synthesis in non-insulin-dependent diabetic mice induced by high-fat diet and streptozotocin. American journal of physiology Heart and circulatory physiology.

[55] Mancini A, Jovanovic DV, He QW, Di Battista JA (2007). Site-specific proteolysis of cyclooxygenase-2: a putative step in inflammatory prostaglandin E(2) biosynthesis. J Cell Biochem.

[56] Liu T, Schneider RA, Shah V, Huang Y, Likhotvorik RI (2009). Protein Never in Mitosis Gene A Interacting-1 regulates calpain activity and the degradation of cyclooxygenase-2 in endothelial cells. J Inflamm (Lond).

[57] Wei X, Zhang X, Flick LM, Drissi H, Schwarz EM (2009). Titanium particles stimulate COX-2 expression in synovial fibroblasts through an oxidative stress-induced, calpain-dependent, NF-kappaB pathway. American journal of physiology Cell physiology.

[58] Triggle CR, Howarth A, Cheng ZJ, Ding H (2005). Twenty-five years since the discovery of endothelium-derived relaxing factor (EDRF): does a dysfunctional endothelium contribute to the development of type 2 diabetes?. Can J Physiol Pharmacol.

[59] Cheng ZJ, Vaskonen T, Tikkanen I, Nurminen K, Ruskoaho H (2001). Endothelial dysfunction and salt-sensitive hypertension in spontaneously diabetic Goto-Kakizaki rats. Hypertension.

